# Effect of plateletpheresis on hematocrit, hemoglobin and erythrocyte count: Meta-analysis 1980–2018

**DOI:** 10.1038/s41598-019-56175-7

**Published:** 2019-12-24

**Authors:** Alejandro Gil-Betacur, Carmen Yulieth Mantilla-Gutiérrez, Jaiberth Antonio Cardona-Arias

**Affiliations:** 1 Microbiólogo y Bioanalisista. MSc Microbiología y Bioanálisis. Grupo de investigación Salud y Sostenibilidad, Escuela de Microbiología, Universidad de Antioquia, Medellín Colombia, Banco de Sangre Universidad de Antioquia, Medellín, Colombia; 2 Bacterióloga y laboratorista Clínica, MSc Microbiología y Bioanálisis, Universidad de Antioquia, Hospital General de Medellín Luz Castro de Gutiérrez, Medellín, Colombia; 3 Microbiólogo y Bioanalisista, MSc Epidemiología, MSc Economía aplicada, PhD (candidato) Salud Pública, Universidad de Antioquia UdeA, Calle 70 No. 52-21, Medellín, Colombia

**Keywords:** Biomarkers, Physics

## Abstract

The effects of platelet donation by apheresis on different parameters of the erythrogram are still unclear. The objective was to meta-analyze the effect of plateletpheresis on hematocrit, hemoglobin, and erythrocyte count, with a systematic review with random effects meta-analysis of the mean difference. The PRISMA guidelines were considered, as well as 133 search strategies on four different databases. Reproducibility was guaranteed and methodological quality was evaluated. Heterogeneity was evaluated with Galbraith and DerSimonian-Laird’s, publication bias with a funnel plot and a Begg’s test, sensitivity analysis and a cumulative meta-analysis were also conducted. Eighteen (18) articles were included, 17 evaluated the effects on hematocrit in 2,564 donors; 13 on hemoglobin in 1,640 donors; and 4 on red blood cell count in 243 donors. A decrease of 2.26% (CI95% = 2.11–2.41) was observed in hematocrit, of 0.80 g/dL (CI95% = 0.75–0.86) in hemoglobin and −0.21 × 10^12^/L (CI95% = −0.13; −0.29) in red blood cell count. Plateletpheresis has a negative effect on the erythrogram parameters, explained by blood loss in the kits used for the procedure and cell lysis. Such evidence is relevant to secure the efficiency and safety of the procedure, improve selection processes or determine the number of donations that can be performed without affecting donors’ health.

## Introduction

The criteria established in each country for donations of blood or its components seek to protect donors and patients, obtain products that meet high-quality standards, and ensure that donations do not result in health problems for donors^1^. In that regard, the procedures by apheresis offer, in comparison with manual blood donations, advantages such as shorter periods of times required to be left between donations^2^, a decrease of adverse events in patients, like refractoriness and alloimmunization, as well as more controlled doses and volume collection.

However, there are reports on the possible adverse effects of apheresis platelet donation. Some of these may be short-term, such as those related with the anticoagulant (ACD-A), and others are associated with the procedure, like the loss of red blood cells; although these are not considered significant due to the current availability of technology^1^, some authors report that plateletpheresis can have long-term consequences such as anemia, iron deficiency and thrombocytopenia^3^. Other authors present opposing data while stating that platelet donation by apheresis does not entail a significant loss of erythrocytes or of any other cell component^1–4^.

In its guide for the selection of donors published in 2012, the World Health Organization recommends measuring hemoglobin before proceeding with blood donations. Since this protein screens anemia but does not show iron reserves^3^ there is a significant risk for recurring donors of developing iron deficiency, just as it was shown in a meta-analysis that resulted in repeating donors having a high prevalence of iron deficiency, which could derive in fatigue, cognitive problems, among other clinical events. These records demonstrate the importance of monitoring donors’ health. However, regarding plateletpheresis, there is no such evidence in the case of repeating donors^5,6^.

The effect of platelet donation on the red cell series has been addressed in several studies with differing results, as shown below. Love^7^ found an increase in hemoglobin (0.52–0.37 g/dL) and hematocrit (1.3–1.1%) after donation; Moog^8^ reported an increase in hematocrit (0.3%) and in red blood cell counts (0.0410^12^/L), but unchanged hemoglobin at the end of the plateletpheresis; Landžo^9^ also reported on increased hematocrit levels in the subgroup going through continuous flow procedures (0.30%), while in the subgroup with discontinuous flow procedures there were no changes in hemoglobin, but a decrease in hematocrit. Other studies that analyzed the effect of platelet donation by apheresis showed a decrease in the studied values, such as the report by Das^10^, which found reductions in hematocrit (2.1%) and hemoglobin (1.3 g/dL), and Kim^11^, whose study resulted in a decrease of hemoglobin between 0.3 and 0.7 g/dL.

The heterogeneity of these reports demonstrated the need to systematize the studies published. Systematic reviews allow the evaluation of a larger amount of research and participants, articulate the information from different regions, provide more precise estimations and comparisons with higher statistical power, and generate better-quality evidence while allowing the observation of trends in the evaluated values, as well as the consolidation or rejection of hypotheses^12^. In this specific case, the purpose was to find evidence of the actual effect of plateletpheresis on blood donors and therefore, facilitate the creation of more efficient donation protocols.

In accordance, the main aim of this research was to meta-analyze the effect of plateletpheresis on hematocrit, hemoglobin and erythrocyte count.

## Methods

### Type of study

Systematic revision of literature with meta-analysis.

### Pico question: population intervention comparison outcome

#### Population

Donors of platelets by apheresis.

#### Intervention

Plateletpheresis

#### Comparison

Pairwise comparison of the difference between measurements before the procedure and the ones immediately or 1 hour later.

#### Outcome

Hematocrit, hemoglobin and red blood cell count through systematic hemogram. It is worth mentioning that during the initial protocol there was an observation of the other erythrogram parameters, but the amount of research found was not enough for a meta-analysis.

### Research and selection of studies using collaboration between Cochrane and PRISMA (Preferred Reporting Items for Systematic reviews and Meta-Analyses)

#### Identification

Research based on sensitivity and specificity in Medline-PubMed, Scielo, Science Direct, and Scopus databases^13^. The search was conducted combining 9 terms related to plateletpheresis donation: plateletpheresis, plateletphereses, phrombocytapheresis, thrombocytaphereses, thrombocytopheresis, thrombocytophereses, platelet apheresis, thrombapheresis, and haemapheresis platelet; and 12 terms related with hemogram and adverse reactions to donations: adverse reactions, iron deficiency, ferritin, hematological (indices, values, parameters), hematological (indices, values, parameters), blood (indices, values, parameters). On the other hand, the search in Spanish was conducted using 5 terms related with platelet donation: plaquetoferesis sanguínea, trombocitaferesis, plaquetas por aféresis, plaquetaferesis, plaquetoféresis; and its combination with 5 terms related with hemogram: reacciones adversas, deficiencia de hierro, ferritina, hemograma, hemoleucograma.

#### Screening

The studies included in the analysis met the following criteria: search terms in title, abstract or keywords, original and longitudinal studies in apheresis donors, published in English, Portuguese or Spanish. There was no time limit established for the search. The time frame was defined between the date of the oldest study in the revision and the last protocol update performed in the second semester of 2018.

Some of the search strategies are presented below, according to the database: PubMed *(Thrombocytapheresis [Title/Abstract] AND hematological indices[Title/Abstract])*, in ScienceDirect Title, *abstract, keywords: Plateletpheresis ferritin*, in Scopus: *TITLE-ABS-KEY (“plateletpheresis”) AND TITLE-ABS-KEY (“haematological parameters”)* and in Scielo: (ab:(Plaquetoferesis AND deficiencia de hierro)).

#### Selection

The studies about acute adverse reactions, about other donations than plateletpheresis, articles not available in the databases and studies that did not evaluate hemoglobin, hematocrit, red blood cell count or any other parameter of the red cell series were excluded.

#### Inclusion

**Figure 1:**
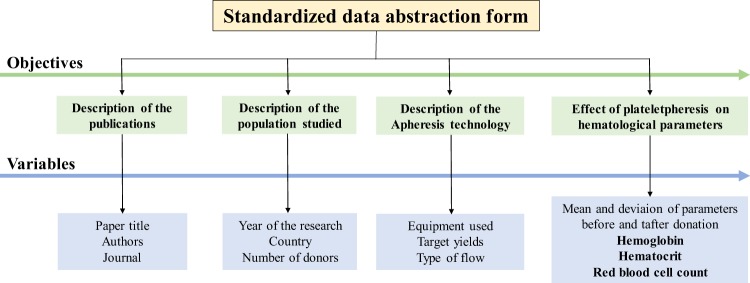
Standardized data abstraction form.

The variables title, author, journal, country, year of publication, number of donors, apheresis technology used and hemoglobin, hematocrit and red blood cell count before and after donation were extracted from the articles that met the protocol (Fig. 1).

### Evaluation of reproducibility and methodological quality

The analysis of the reproducibility of the search and selection of articles, as well as the extraction of the variables, was made by two researchers that applied the protocol. The evaluation of the methodological quality was made according to the STROBE (*Strengthening the Reporting of Observational Studies in Epidemiology*) statement^14^, despite this being an editorial guide that contains criteria for the evaluation of internal and external validity of observational research.

### Analysis of information

the studies were described using frequencies. Heterogeneity was evaluated using a Galbraith graphic, DerSimonian-Laird’s statistic (Q statistic with chi-squared distribution) and RI coefficient (Proportion of the total variance in response to between-study variance). Publication bias was evaluated using a funnel plot and a Begg’s test (Z statistic). A sensitivity analysis was conducted, together with an influence diagram. The results were shown through a forest plot and a cumulative meta-analysis. The analysis was conducted using EPIDAT 3.1.

## Results

**Figure 2:**
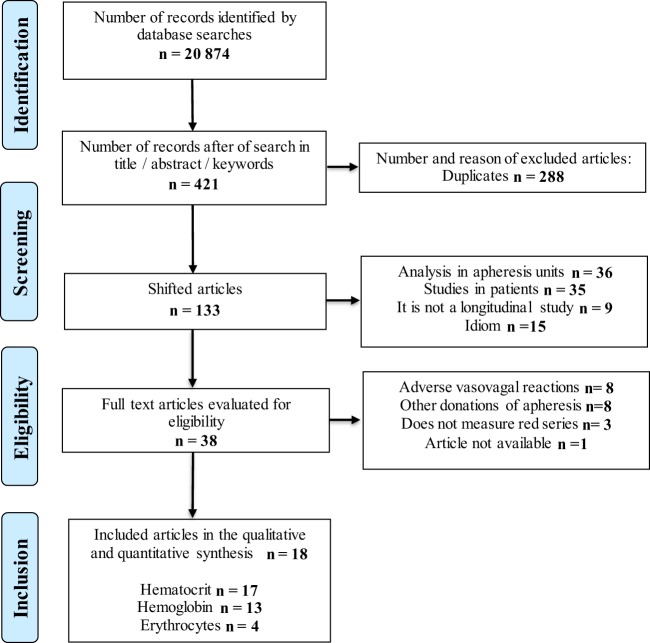
Flow gram of search and selection of studies.

After using the search and study selection protocol without any filters, 20,874 studies were found; the articles decreased to 133 studies when selected according to the title, abstract or keyword and after eliminating duplicates. After inclusion and exclusion criteria were applied, 18 articles were included for qualitative and quantitative synthesis 17 articles that evaluate the effect of platelet donation on the hematocrit, 13 on the hemoglobin and 4 on the red blood cell count (Fig. 2).

The countries with the highest frequency of studies were India, with 5 studies, Turkey, with 3, Germany and Austria with 2 studies each. Forty-four percent (44%) of the studies was published between 1980 and 2008, 17% in 2009, and the remaining 39% between 2010 and 2017. In total, 2,614 plateletpheresis donors were included. In some studies, the analysis was conducted by subgroups according to the equipment employed, the quantity of collected product, separation technique or gender (Table 1).

**Table 1:** Description of the studies according to year, country, population and parameter analyzed.

Author	Year	Country	N	Hematocrit (%) Mean ± Desviation		Hemoglobin (g/dL) Mean ± Desviation		Erythrocytes (×10^12^ L) Mean ± Desviation	
				Pre	Post	Pre	Post	Pre	Post
Katz^4^	1980	U.S.	24	42.2 ± 2.2	40 ± 2.3	14.1 ± 0.7	13.3 ± 0.8	N/A	N/A
Rock^25^	1992	Canada	13	44.0 ± 2.0	43 ± 2.0	14.7 ± 0.7	14.3 ± 0.7	N/A	N/A
Love^7^	1993	Britain	78^a^	41.8 ± 2.2	43.1 ± 2.4	14.7 ± 0.7	15.2 ± 0.9	N/A	N/A
			34^b^	38.9 ± 2.4	40.0 ± 2.9	13.6 ± 0.9	13.9 ± 1.0	N/A	N/A
Buchholz^26^	1997	U.S.	26	40.0 ± 3.0	36.0 ± 3.0	13.3 ± 1.0	12.0 ± 1.0	N/A	N/A
Beyan^27^	2003	Turkey	265	43.7 ± 2.8	41.2 ± 2.8	14.9 ± 1.0	14.0 ± 1.0	N/A	N/A
Ifran^28^	2005	Turkey	35	46.4 ± 3.0	42.9 ± 3.6	15.1 ± 0.8	14.2 ± 1.2	5.2 ± 0.3	4.9 ± 0.5
Bor^29^	2008	Turkey	20	45.1 ± 3.07	45.8 ± 2.9	14.7 ± 0.7	14.4 ± 0.8	N/A	N/A
Kim^11^	2008	South Korea	25^c^	N/A	N/A	14.5 ± 0.7	13.8 ± 2.1	N/A	N/A
			25	N/A	N/A	15.5 ± 1.1	15.7 ± 1.1	N/A	N/A
Moog^8^	2009	Germany	60	42.6 ± 3.0	42.9 ± 3.6	14.6 ± 1.0	14.6 ± 1.6	4.7 ± 0.4	4.7 ± 0.4
Das^10^	2009	India	457	40.8 ± 4.0	38.9 ± 3.4	13.9 ± 1.1	12.6 ± 4.74	N/A	N/A
Tendulkar^30^	2009	India	121^d^	41.6 ± 3.5	40.6 ± 3.3	13.7 ± 1.0	13.4 ± 1.1	N/A	N/A
			50^e^	41.4 ± 2.8	39.2 ± 3.0	13.6 ± 1.0	12.9 ± 1.1	N/A	N/A
			66^f^	43.0 ± 2.6	41.7 ± 3.1	14.1 ± 0.9	13.7 ± 1.1	N/A	N/A
Wan^31^	2011	Malaysia	76	44.6 ± 2.5	44.1 ± 2.6	14.9 ± 0.9	14.7 ± 1.0	N/A	N/A
Macher^32^	2012	Austria	24^g^	44.9 ± 2.9	40.9 ± 2.9	15.4 ± 1.3	14.1 ± 1.3	5.2 ± 0.4	4.7 ± 0.4
			24^h^	44.4 ± 2.6	40.8 ± 3.1	15.2 ± 1.1	13.9 ± 1.3	5.1 ± 0.4	4.7 ± 0.45
Heuft^33^	2012	Germany -Austria	185^i^	43.4 ± 3.3	42.5 ± 7.0	14.5 ± 1.1	14.2 ± 2.3	N/A	N/A
			226^j^	43.2 ± 3.3	43.3 ± 4.0	14.4 ± 1.1	14.4 ± 1.3	N/A	N/A
Patidar^34^	2012	India	500	40.2 ± 1.7	36.2 ± 2.3	13.3 ± 0.6	12.1 ± 0.8	N/A	N/A
Nomani^35^	2013	India	60	43.9 ± 2.6	41.2 ± 2.7	13.4 ± 0.8	12.4 ± 0.8	N/A	N/A
Gite^36^	2015	India	100	40.9 ± 2.4	39.8 ± 2.7	13.7 ± 1.2	12.9 ± 1.2	5.1 ± 0.5	4.9 ± 0.6
Landžo^9,36^	2017	Croatia	60^k^	43.4 ± 2.7	43.7 ± 2.6	15.4 ± 1.0	14.6 ± 1.1	N/A	N/A
			60^l^	44.4 ± 2.8	41.3 ± 2.8	14.3 ± 1.0	14.3 ± 1.0	N/A	N/A

^a^Men. ^b^Woman, ^c^Control, ^d^Amicus, ^e^Fenwal CS-3000 Plus, ^f^Cobe spectra. ^g^Fenwal Amicus, ^h^Caridian BCT Trima Accel. ^i^Double plateletheresis, ^j^Triple plateletheresis. ^k^Continuous Flow, ^l^Intermittent Flow.

**Figure 3:**
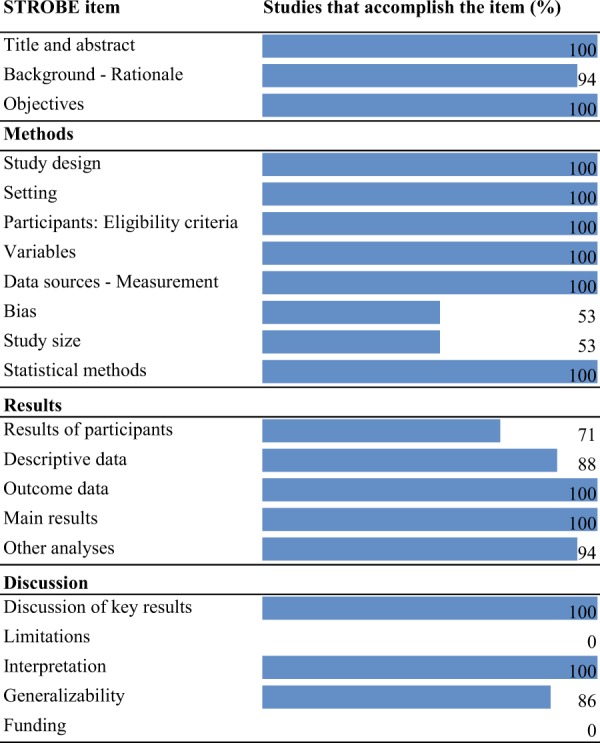
Evaluation of methodological quality.

After evaluating methodological quality, the study by Love^5^ met the fewest criteria, with 68% of fulfillment. The rest of the studies met more than 72% of the items in the STROBE statement. Neither the funding source nor the statement of limitations was specified in any of the studies; besides, only 53% reported the source of bias, and 53%, the calculation of the sample size (Fig. 3).

**Figure 4:**
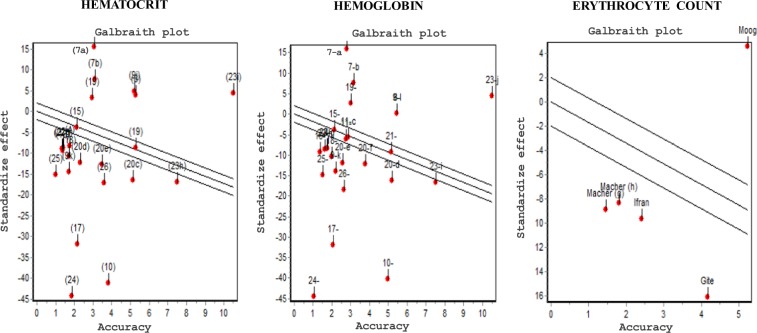
Analysis of heterogeneity with Galbraith.

**Figure 5:**
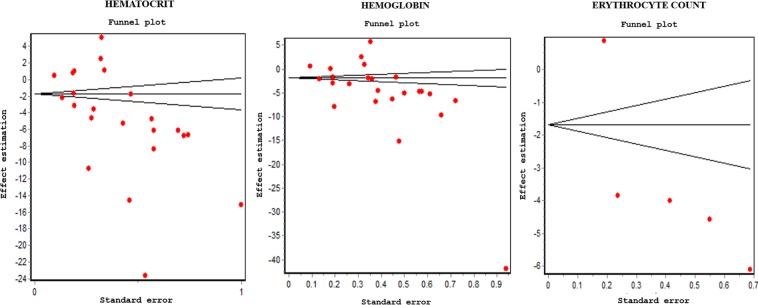
Evaluation of publication bias with Funnel Plot.

High heterogeneity was found in hematocrit, hemoglobin and erythrocyte count (Fig. 4). The DerSimonian-Laird Q statistic had p = 0,000 and RI coefficient = 99%. There was no publication bias (Fig. 5) and the influence diagram showed that the elimination of a study does not alter the direction nor the significance of the global effect, evidencing thus the excellent sensitivity of the combined measure.

The global meta-analysis through a random effects model showed a mean difference (pre and post-donation) of −2.26% with CI95% 2.11–2.41% (Z = 29.8, p < 0.001) in hematocrit; −0.80 g/dL with CI 95% 0.75–0.86 g/dL (Z = 29.0, p < 0.001) in hemoglobin; and −0.21 × 10^12^/L with CI95% −0.13; −.29 × 10^12^/L (Z = 5.23, p < 0.001) in erythrocyte count after the procedure. The cumulative meta-analysis showed a statistically significant difference (negative effect) based on 1,133 donors in hematocrit, 1,170 donors in hemoglobin, and 243 donors in erythrocyte count which demonstrates that the effect of plateletpheresis was not detected in small-sized samples, and the importance of ensuring high statistical power in this type of comparisons.

## Discussion

This meta-analysis entailed the systematization of 18 studies that evaluated the effect of platelet donation by apheresis on hematocrit, hemoglobin or erythrocyte count, in a total of 2,564, 1,640 and 243 donors respectively. This large sample size facilitated more precise estimation of the effects assessed, a higher possibility of extrapolation of results, higher statistical power in comparisons, among other advantages of this type of studies^12^. In the specific case of this research, the sample size demonstrates the importance of having evidence with good statistical power to evaluate the effect of apheresis procedures.

The highest frequency of publications was found in India. Since hemoglobinopathies are the most common hereditary disorder in this country, with 30 million people carrying the disease and requiring recurring blood transfusions as an essential part of their treatment^15,16^, the existence of a clinical and epidemiological link could be implied, justifying the existing interest in research on the effects of this type of processes. In this sense, the scarcity of studies about the subject in Latin America is a point of interest given the number of patients that frequently and constantly require transfusional support in the region. The situation demands the analysis of the changes that apheresis donors may present once the procedure is over, in order to ensure their safety and hence, constant availability of blood components.

Seventy-eight (78%) of studies were published after 2000, which shows that there is a recent interest in the state of apheresis donors after the procedure. In countries like Colombia, as this type of donation has become more frequent in the recent years, unmet demand has decreased from 10.9% in 2015^17^ to 9.8% in 2016^18^. This demonstrates both the current importance of apheresis donation and the need for research focused on its safety, given that it is one of the procedures that may be most often conducted in transfusional medicine.

The three variables showed heterogeneous results, maybe due to the different studied populations, as well as the number of donations by each donor, the equipment used for donations or even the year of the analysis since the first equipment employed used to cause higher cell loss^1^. Despite the importance of the variables as reasons to explain the heterogeneity found, it is necessary to mention that the reports on individual studies did not allow meta-regressions or analyses with this type of subgroups, which could be conducted in future studies.

Despite this heterogeneity, the sensitivity analysis showed that none of the studies affected the combined measure, demonstrating the validity of the measure estimated through the random effects model.

The results of the forest plot show that platelet donation by apheresis causes hematocrit, hemoglobin, and erythrocyte count values to decrease, all parameters directly interrelated, especially in healthy individuals as donors are supposed to be. Conversely, the evaluation of the group in Tondon^19^ shows that blood loss in apheresis procedures is small and insignificant; it even proposes that the 20–30 mL of blood loss that the procedure entails is not significant in comparison with total blood donations, which lead to the loss of approximately 450 mL. Additionally, regarding hemoglobin, this means the possibility of decreasing 1 g/dL the selection criteria for this type of donation, in view of its high safety levels.

Despite the controversy explained above, the decrease reported in this meta-analysis may be due to two main reasons. Firstly, blood remnants in the kit’s nozzle amount to 20–45 mL^20,21^ depending on the equipment employed. Secondly, when erythrocytes are exposed to stress or changes in the osmotic pressure, there may be hemolysis or rupture in the extracorporeal circulation that takes place during the procedure.

Moreover, as Schotten^22^ says, in the first four days post-donation there is an evident decrease of hemoglobin due to physiological compensation that takes place to make up for the loss of blood volume that generates a dilutive effect. It is also worth noting that during apheresis procedures, donors are exposed to saline solutions and anticoagulant infusions that could aggravate this effect. These two explanations would entail the need to deepen research related to the time to compensate such loss or the normalization of the physiological process described, in order to ensure donor’s safety.

It must be clarified that in clinical or physiological terms, such loss corresponds to short-term evaluations (immediately after or 1 hour after the procedure), which could be normalized or compensated in periods of time that are the same as or shorter than those recommended in the protocol for subsequent platelet donation by apheresis.

Future studies should evaluate the effect of the procedure on ferritin, which is better than hemoglobin^23^ to indicate the state of iron reserves for future donations. Such suggestion stems from the revision of diverse studies in total blood donors^22,24^ which concluded that donors with low ferritin do not reach their pre-donation levels back, and erythropoiesis is not apt due to a reticulocyte maturation process, perhaps caused by reduced iron bioavailability.

The effect on the other erythrogram parameters and iron metabolism indicators could also be evaluated to observe all the effects of the procedure on erythrocytes and improve measures before donation if necessary. Accordingly, the red cell series could be measured in first-time donors to gain insight into changes suffered in donors without initial loss or determine the effect of recurrent donations.

The absence of physiological data about donors, number of previous donations, or reports on other variables can be mentioned among the limitations of this research. Another limitation lies in the fact of not being able to perform meta-regressions by variables such as equipment used, target yields, and flow technology given the low number of studies that reported the values of the hematic parameters by these variables. These do not allow the identification of the source of the heterogeneity of the studies nor the ensuing explanation of the net effect of the procedure on decreased hematocrit, hemoglobin, and erythrocytes.

## Conclusion

Plateletpheresis has a negative effect on the parameters of the erythrogram, explained by blood loss in the kits employed in the procedure and cell lysis. Such evidence is relevant to improve selection processes and efficiency and safety in the procedure or establish the number of donations that can be made without affecting donors’ health.

## Data Availability

All the data are available in the article.
